# Altered Ca^2+^ and Na^+^ Homeostasis in Human Hypertrophic Cardiomyopathy: Implications for Arrhythmogenesis

**DOI:** 10.3389/fphys.2018.01391

**Published:** 2018-10-16

**Authors:** Raffaele Coppini, Cecilia Ferrantini, Alessandro Mugelli, Corrado Poggesi, Elisabetta Cerbai

**Affiliations:** ^1^Department of Neuroscience, Psychology, Drug Sciences and Child Health (NEUROFARBA), University of Florence, Florence, Italy; ^2^Department of Experimental and Clinical Medicine, University of Florence, Florence, Italy

**Keywords:** cardiac hypertrophy, ion channels, ranolazine, arrhythmias, afterdepolarization, calmodulin kinase (CaMKII), T-tubules, beta adrenergic

## Abstract

Hypertrophic cardiomyopathy (HCM) is the most common mendelian heart disease, with a prevalence of 1/500. HCM is a primary cause of sudden death, due to an heightened risk of ventricular tachyarrhythmias that often occur in young asymptomatic patients. HCM can slowly progress toward heart failure, either with preserved or reduced ejection fraction, due to worsening of diastolic function. Accumulation of intra-myocardial fibrosis and replacement scars underlies heart failure progression and represents a substrate for sustained arrhythmias in end-stage patients. However, arrhythmias and mechanical abnormalities may occur in hearts with little or no fibrosis, prompting toward functional pathomechanisms. By studying viable cardiomyocytes and trabeculae isolated from inter-ventricular septum samples of non-failing HCM patients with symptomatic obstruction who underwent myectomy operations, we identified that specific abnormalities of intracellular Ca^2+^ handling are associated with increased cellular arrhytmogenesis and diastolic dysfunction. In HCM cardiomyocytes, diastolic Ca^2+^ concentration is increased both in the cytosol and in the sarcoplasmic reticulum and the rate of Ca^2+^ transient decay is slower, while the amplitude of Ca^2+^-release is preserved. Ca^2+^ overload is the consequence of an increased Ca^2+^ entry via L-type Ca^2+^-current [due to prolongation the action potential (AP) plateau], combined with a reduced rate of Ca^2+^-extrusion through the Na^+^/Ca^2+^ exchanger [due to increased cytosolic (Na^+^)] and a lower expression of SERCA. Increased late Na^+^ current (I_NaL_) plays a major role, as it causes both AP prolongation and Na^+^ overload. Intracellular Ca^2+^ overload determines an higher frequency of Ca^2+^ waves leading to delayed-afterdepolarizations (DADs) and premature contractions, but is also linked with the increased diastolic tension and slower relaxation of HCM myocardium. Sustained increase of intracellular [Ca^2+^] goes hand-in-hand with the increased activation of Ca^2+^/calmodulin-dependent protein-kinase-II (CaMKII) and augmented phosphorylation of its targets, including Ca^2+^ handling proteins. In transgenic HCM mouse models, we found that Ca^2+^ overload, CaMKII and increased I_NaL_ drive myocardial remodeling since the earliest stages of disease and underlie the development of hypertrophy, diastolic dysfunction and the arrhythmogenic substrate. In conclusion, diastolic dysfunction and arrhythmogenesis in human HCM myocardium are driven by functional alterations at cellular and molecular level that may be targets of innovative therapies.

## Introduction: Arrhythmic Substrate in HCM, from Tissue to the Single Myocyte

Hypertrophic cardiomyopathy (HCM) is the most common monogenic inheritable heart disease (Maron et al., 2000; Gersh et al., 2011; Authors/Task Force Members et al., 2014), with a prevalence of 1:500. HCM is a leading cause of sudden cardiac death in the young (Maron et al., 2000) and a prevalent cause of heart failure and stroke in all age groups (Maron et al., 2003). Mutations in genes coding for sarcomeric proteins are found in over 60% of patients with HCM, the most commonly involved genes being *MYH7* (β-myosin heavy-chain) and *MYBPC3* (cardiac myosin-binding protein-C). Increased ventricular arrhythmogenesis is one of the main pathophysiological features of this disease (Olivotto et al., 2009) and is responsible for the heightened risk of lethal arrhythmias in HCM patients. Despite being only mildly symptomatic in about 2/3 of patients (Maron et al., 2016), HCM can slowly progress toward heart failure, either with preserved or reduced ejection fraction, due to worsening of diastolic and/or systolic function (Olivotto et al., 2012). Accumulation of intra-myocardial fibrosis and replacement scars underlies heart failure progression and represents a substrate for sustained arrhythmias (Galati et al., 2016). Late gadolinium enhancement (LGE) at cardiac magnetic resonance, an index of cardiac fibrosis, is widely used to stratify the severity of disease progression (Authors/Task Force Members et al., 2014) and to help defining the risk of lethal arrhythmias and terminal heart failure (Chan et al., 2014). However, LGE only identifies extensive replacement scars and well predicts the risk of arrhythmias only at the advanced stages of the disease (Chan et al., 2014). Replacement fibrosis in HCM appears to be related with local myocardial ischemia, caused by microvascular dysfunction (Sotgia et al., 2008), as myocardial tissue in regions with severe microvascular ischemia is slowly replaced by collagen. The degree of microvascular dysfunction, as assessed by positron emission tomography with labeled ammonia measuring the reduction of local myocardial blood flow, is related with patient outcome, including the risk of arrhythmias at advanced disease stages (Cecchi et al., 2003). Replacement fibrosis and microvascular ischemia are strongly related with the risk of ventricular arrhythmias because they create a stable substrate for reentry circuits, the main drivers of sustained ventricular arrhythmias (Pogwizd and Corr, 1987). In order for a reentry circuit to be established, an area of conduction block adjacent to a region of slower, unidirectional conduction is needed: indeed, in HCM myocardium, patchy replacement fibrosis generates areas of no-conduction, while the surrounding ischemic regions (due to microvascular dysfunction) cause slower, altered conduction, and transient local alterations of repolarization (Hurtado-de-Mendoza et al., 2017). Following these observation, the simplest conclusion would be that structural left ventricular remodeling at macroscopic myocardial level, featuring replacement fibrosis and microvascular dysfunction, is the main determinant of arrhythmias in HCM. However, a clear relationship between fibrosis (measured with LGE), microvascular dysfunction (measured by PET) and arrhythmic risk is observed only in the minority of patients (10–15%) that experience a slow progression toward end-stage HCM, an highly arrhythmogenic condition not unlike terminal ischemic heart failure, thus requiring aggressive preventive strategies (Priori et al., 2015). On the contrary, the majority of sudden cardiac death events due to lethal ventricular arrhythmias occur in patients at earlier stages of disease progression, often in the absence of marked structural abnormalities besides left-ventricular (LV) hypertrophy, also in very young patients (Maurizi et al., 2018). In early stages of hypertrophic cardiomyopathies, replacement scars are absent and only microscopic intramyocardial fibrosis is present, and its extent can be assessed by CMR using T1-mapping (Dass et al., 2012) or extracellular volume (ECV) measurements with gadolinium contrast, both altered even before the onset of hypertrophy in HCM-mutation carriers (Ho et al., 2013). However, the link between the degree of ECV expansion and the risk of arrhythmias in early stage HCM is still uncertain (Avanesov et al., 2017). Therefore, arrhythmias in HCM cannot be the sole consequence of the substrate for re-entry circuits at tissue level. On the contrary, the main determinants of arrhythmogenesis in HCM are to be found within the affected cardiomyocytes, a consequence of the alterations of ion currents and intracellular Ca^2+^ handling that occur as a part of HCM-related ventricular cardiomyocyte remodeling (Coppini et al., 2013; Coppini et al., 2017; Ferrantini et al., 2017; Ferrantini et al., 2018). Indeed, from a pathophysiological standpoint, the vast majority of ventricular tachycardia episodes begin with one or more premature ectopic ventricular beats (Ulus et al., 2013). Premature ventricular activations are essential to initiate the abnormal rotating conduction of the re-entry circuits in the presence of an appropriate substrate. Even in the presence of extended structural alterations, such as large scars and diffuse interstitial fibrosis, ectopic premature activations initiating in abnormal cardiomyocytes could be essential triggers to initiate the re-entry that is then maintained by the structural substrate. Interestingly, ectopic activity is the primary event producing local unidirectional block, which is an essential prerequisite for the establishment of a re-entry circuit. Ectopic ventricular beats are very common in HCM patients (Adabag et al., 2005) and originate from the premature spontaneous premature activation of a group of adjacent synchronized ventricular cardiomyocytes within the ventricular mass, which is then propagated to the whole surrounding ventricular mass, often in a chaotic and unpredictable manner (Sato et al., 2009). Early- and delayed- afterdepolarizations are the cellular arrhythmic events that may result into the spontaneous generation of premature action potentials in the affected cardiomyocytes. The following part of this review will illustrate how an increased likelihood of early and delayed after-depolarizations in HCM cardiomyocytes is a consequence of specific alterations of ion currents and intracellular Ca^2+^-handling.

## Materials and Methods

Most of the results presented in the figures were previously published in Coppini et al. (2013) and in Ferrantini et al. (2018). Previously unpublished results are presented in **Figures 3, 4**. Cardiomyocytes and intact trabeculae were freshly isolated as previously described (Coppini et al., 2013; Ferrantini et al., 2018), using surgical upper inter-ventricular septum samples excised from HCM patients who underwent myectomy operation for the relief of severe symptoms due to obstruction of the LV outflow tract. Notably, all recruited patients had preserved systolic function but impaired diastole and most of them have a history of documented non-sustained ventricular tachycardia at Holter monitoring. The experimental protocols were approved by the ethical committee of Careggi University-Hospital of Florence (2006/0024713, renewed May 2009; 2013/0035305). In single cardiomyocytes, APs and Ca^2+^ transients were simultaneously measured using combined patch-clamp and Ca^2+^-fluorescence measurements, during stimulation at different frequencies. Cell capacitance was measured in voltage-clamp mode. Isometric force was measured from intact trabeculae during electrical field stimulation. Cells/trabeculae were exposed to test drugs for at least 5 min prior to recordings. Number of cells/trabeculae for each comparison, as well as the statistical tests used, are indicated in the respective figure legends.

## Remodeling of Ion Currents in HCM Cardiomyocytes

**FIGURE 1:**
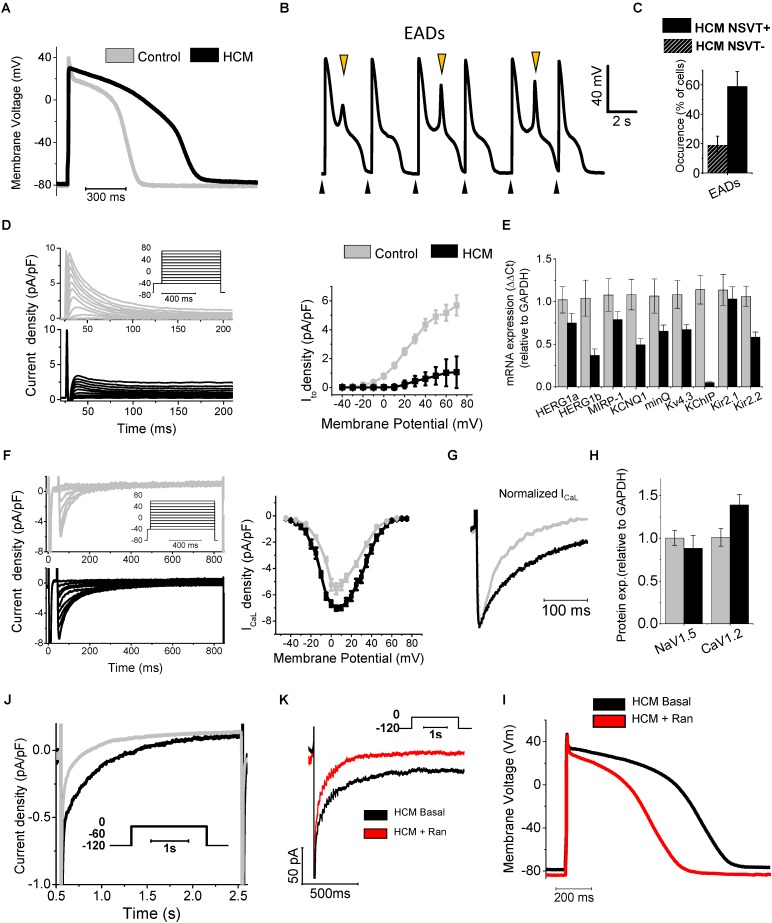
Remodeling of ion currents in HCM cardiomyocytes **(A)** Superimposed representative action potentials recorded during stimulation at 0.2 Hz from HCM and control cardiomyocytes. **(B)** Representative recording from an HCM cardiomyocyte paced at 0.2 Hz, showing EADs. Black arrows mark stimuli. Orange arrows mark EADs. **(C)** Occurrence of EADs in 23 cells from patients without NSVT (NSVT-) and 29 from patients with NSVT (NSVT+). **(D)** Representative traces (left, top panel control, bottom panel HCM) and average I_to_ activation curves from control and HCM cardiomyocytes (right panel). **(E)** RNA expression of potassium current genes, relative to GAPDH in control (*N* = 11, gray) and HCM (*N* = 15, black) samples. **(F)** Left: representative I_CaL_ traces at different voltages. Right: I_CaL_ activation curves. **(G)** Superimposed normalized I_CaL_ recordings at 0 mV. **(H)** Protein expression of I_CaL_ and I _Na_ main channel subunits (Cav1.2 and Nav1.5). **(J)** Representative I_NaL_ traces from control and HCM cardiomyocytes. **(K)** Ranolazine inhibits I_NaL_ in HCM cardiomyocytes: representative I_NaL_ traces from an HCM cardiomyocyte during depolarization to -20 mV in the absence (Basal) or presence of Ran. **(I)** Action potentials at 0.2 Hz from an HCM cardiomyocyte before (Basal) and after exposure to 10 μmol/L ranolazine (Ran). Modified from Coppini et al., 2013 (ref. 19).

Hypertrophic cardiomyopathy (HCM) cellular pathophysiology is characterized by the interplay between primary alterations (direct consequences of causal sarcomeric mutations altering myofilament function) and a large number of secondary myocardial modifications, comprising cellular electrophysiological remodeling (changes in transmembrane currents) and alterations of intracellular Ca^2+^ handling [Ca^2+^ transients and diastolic (Ca^2+^)] (Coppini et al., 2013; Coppini et al., 2017; Ferrantini et al., 2017; Ferrantini et al., 2018). Due to the profound electrophysiological differences between mouse and human cardiomyocytes, studies conducted on transgenic rodent models did not help to identify the features if ion-current remodeling occurring in HCM myocardium (van der Velden et al., 2015). To overcome this limitation, we investigated the abnormalities of electrical function, Ca^2+^ handling and contraction of human HCM myocardium as coexisting contributing factors for contractile dysfunction and arrhythmias in this disease (Coppini et al., 2013). We used isolated myocytes and intact trabeculae from fresh myocardial samples from the interventricular septum of HCM patients undergoing surgical myectomy operation for refractory symptoms related to severe obstruction of the left ventricular outflow tract, compared with septal samples from non-hypertrophic surgical patients (Coppini et al., 2013; Ferrantini et al., 2018). Overall, we performed patch-clamp and ion-fluorescence studies in over 200 cardiomyocytes from 43 HCM patients (Coppini et al., 2013; Ferrantini et al., 2018). Results of patch clamp studies in isolated ventricular cardiomyocytes showed that the duration of action potentials (APD), recorded at various pacing rates, was significantly prolonged in cardiomyocytes from HCM cardiomyocytes, with regards to controls (**Figure 1A**). As expected, prolongation of APD was associated with prolonged QTc in patients from the HCM cohort (average QTc = 470 ms). Of note, a recent large multi-center observational study on HCM patients suggested that mild QT prolongation is a common observation in those patients (Johnson et al., 2011). Prolonged APD was the main determinant of the increased risk of early afterdepolarisations (EADs) (Antzelevitch and Belardinelli, 2006), that is, spontaneous depolarisations occurring before the end of the repolarization phase: the occurrence of this type of cellular arrhythmias was 6-fold more frequent in HCM vs. control cardiomyocytes (**Figure 1B**). Interestingly, the frequency of EADs and the degree of APD prolongation went hand in hand with the incidence of ventricular arrhythmias in patients: patients whose cells show markedly prolonged APDs had a higher rate of documented non-sustained ventricular tachycardia at 24 h ECG (Coppini et al., 2013; **Figure 1C**). APD prolongation in HCM cardiomyocytes is caused by a combination of decreased repolarizing potassium currents and increased depolarizing (Ca^2+^ and Na^+^) currents: transient outward K^+^ current (I_to_), inward-rectifier current (I_K1_), as well as delayed rectifier K^+^ currents were significantly reduced, while both L-Type Ca^2+^ current (I_CaL_) and late Na^+^ current (I_NaL_) were increased in HCM cells, as compared to control cardiomyocytes (Coppini et al., 2013; **Figures 1D–H**). Pathological changes of the density of ion currents in HCM cardiomyocytes were caused by different mechanisms. The reduced density of potassium currents in HCM cardiomyocytes was the consequence of the lower levels of expression of K^+^ channel genes (**Figures 1D,E**), as observed in several human and animal models of cardiac hypertrophy and heart failure (Ravens and Cerbai, 2008), including heart failure with preserved ejection fraction (HFpEF) (Cho et al., 2017). In line with other models of LV hypertrophy and diastolic dysfunction (Cho et al., 2017), in human HCM myocardium the ion-channel genes with the most severely depressed expression were I_to_ and I_K1_ (Coppini et al., 2013): this might be a consequence of the increased activity of Ca^2+^/calmodulin-dependent protein-kinase II (CaMKII) in HCM cardiomyocytes (Coppini et al., 2013), which is able to down-regulate I_to_ and I_K1_ currents by reducing the expression of functional channels (Wagner et al., 2009). Moreover, the down-regulation of I_K1_ (Kir2.1) may also be related with the increased expression of micro-RNA miR-1 (Yang et al., 2007) observed in HCM specimens (Coppini et al., 2013). The small increase of I_CaL_ density in HCM cardiomyocytes is likely determined by the slightly higher expression of CACNA1.2 gene and CaV1.2 protein (**Figures 1F–H**). In addition, the inactivation kinetics of I_CaL_ is markedly slower in HCM vs. control cardiomyocytes (Coppini et al., 2013; Ferrantini et al., 2018; **Figure 1G**), contributing to prolong I_CaL_ activation during the AP plateau, thus delaying repolarization. Interestingly, we found that the slower inactivation of I_CaL_ observed in HCM cardiomyocytes may be related with the increased phosphorylation of L-type Ca^2+^ channel β-subunit by CaMKII (Hudmon et al., 2005), observed in HCM myocardium (**Figure 3**). Finally, I_NaL_ was consistently increased in all studied HCM myocytes: I_NaL_ integral (estimating the total Na^+^ flow upon a single current activation) was 2–3 times larger in HCM as compared with control cells (**Figure 1J**). As I_NaL_ is the slowly inactivating or non-inactivating component of Na^+^ current that remains active as a depolarizing current during the AP plateau, it directly contributes to prolong APD in HCM cardiomyocytes, as previously shown in human and animal models of cardiac hypertrophy and heart failure (Maltsev et al., 1998; Pieske and Houser, 2003; Pogwizd et al., 2003). The aforementioned changes in Ca^2+^, late Na^+^ and K^+^ current densities were quantitatively introduced into validated mathematical models of human ventricular myocyte (Grandi et al., 2010; Coppini et al., 2013; Passini et al., 2016): these studies confirmed that the observed ion current changes are sufficient to explain the prolongation of APD in human HCM cardiomyocytes. Also, modeling studies suggested that increased I_NaL_ plays a pivotal role as determinant of APD prolongation and EADs in HCM (Passini et al., 2016), as repolarization reserve is extremely reduced by the down-regulation of K^+^ currents. In support of this hypothesis, we studied the effects of I_NaL_ inhibition by ranolazine (Antzelevitch et al., 2004) or GS-967 (Sicouri et al., 2013) (a potent and selective I_NaL_ blocker) in HCM cardiomyocytes (Coppini et al., 2013; Ferrantini et al., 2018): I_NaL_ inhibition significantly reduced APD by approximately 30% in all HCM cardiomyocytes (**Figure 1**). Of note, ranolazine (at the clinically relevant concentration of 10 μM) and GS-967 (at 0.5 μM) reduced I_NaL_ by 70% in HCM cardiomyocytes. Consistently, the likelyhood of EADs in HCM cardiomyocytes was nearly halved by ranolazine or GS-967. In agreement with the increased role of I_NaL_ as a determinant of APD in the presence of an impaired repolarization reserve, the efficacy of ranolazine in shortening APD was more pronounced at low pacing frequencies and in cells with a longer APD at baseline. Studies in a mathematical cardiomyocyte model (Passini et al., 2016) confirmed that inhibition of 70% of I_NaL_ in HCM myocytes is sufficient to reduce APD, abolish EADs and reduce APD dispersion, as experimentally observed with pharmacological blockers.

## Remodeling of Ca^2+^ Handling in HCM Cardiomyocytes

**FIGURE 2:**
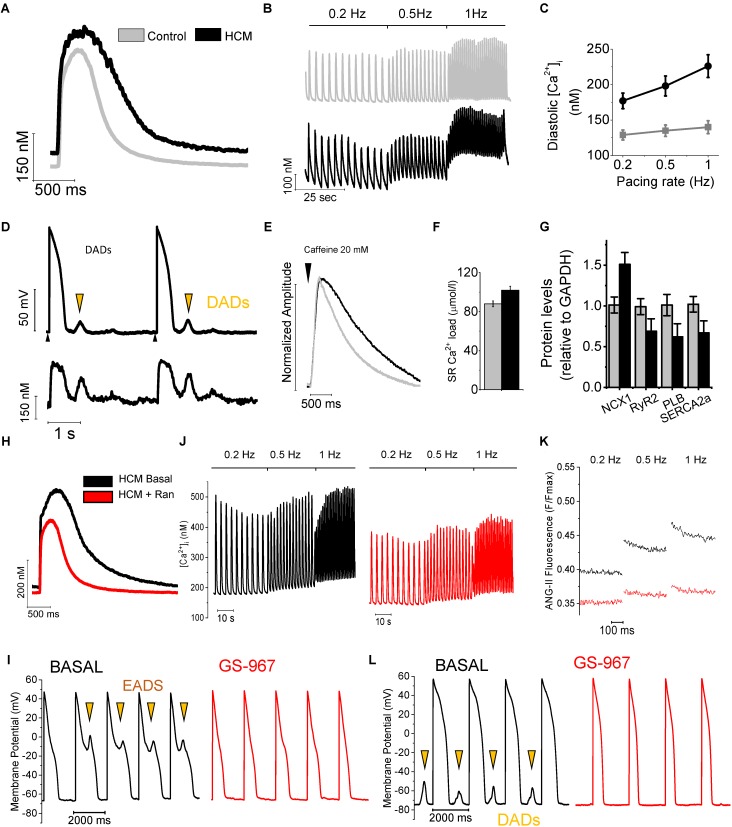
Remodeling of Ca^2+^ handling in HCM cardiomyocytes. **(A)** Superimposed representative Ca^2+^_i_ transients recorded during stimulation at 0.2 Hz. from control and HCM cardiomyocytes. **(B)** Continuous recordings of Ca^2+^_i_ transients elicited at 0.2, 0.5, and 1 Hz frequency of stimulation, from a control (above) and an HCM (below) cardiomyocyte. **(C)** Average diastolic Ca^2+^_i_ fluorescence levels at increasing pacing frequencies. **(D)** Representative recording from an HCM cardiomyocyte, showing DADs, occurring in response to spontaneous Ca^2+^ release (calcium waves). Black arrows mark stimuli. Orange arrows mark DADs. **(E)** Ca^2+^ fluorescence traces during the protocol to measure SR Ca^2+^ content with caffeine: superimposed normalized caffeine-induced calcium transients from HCM and control cardiomyocytes. **(F)** SR Ca^2+^ content (left) in control and HCM cardiomyocytes. **(G)** Protein expression of NCX1, SERCA2a, RYR2 and PLB in HCM (*N* = 10) and control (*N* = 10) specimens. **(H)** Superimposed representative Ca^2+^_i_ transients at 0.2 Hz from an HCM cardiomyocyte at baseline and with ranolazine. **(J)** Trains of Ca^2+^_i_ transients elicited at 0.2, 0.5, and 1 Hz in an HCM myocyte before (left) and following (right) exposure to Ran. **(K)** Representative traces of ANG-II florescence (intracellular sodium) during diastole, recorded at steady state stimulation of 0.2, 0.5, and 1 Hz in the absence (black) and presence (red) of 10 μM Ran. **(I)** Representative trains of action potentials elicited at 0.5 Hz at baseline (black traces) and in the presence of GS-967 0.5 μM (red traces). Early after-depolarizations (EADs) are marked by arrows. **(L)** Representative trains of action potentials elicited at 0.5 Hz. Delayed after-depolarizations (DADs) are marked by arrows. Modified from Coppini et al. (2013) and from Ferrantini et al. (2018).

Notwithstanding the large variabilities observed among different disease models, the common feature that is described in all transgenic animal models, human samples and cellular models of HCM is the sustained increase of diastolic intracellular calcium concentration [(Ca^2+^)]_i_ within ventricular cardiomyocytes (Knollmann et al., 2003; Haim et al., 2007; Ashrafian et al., 2011; Fraysse et al., 2012; Lan et al., 2013). The increase of [Ca^2+^]_i_ is likely to be a direct consequence of some of the sarcomere mutations that cause the disease and thus it may among the first pathological changes in the hearts of HCM-mutation carriers. Indeed, the majority of HCM-related mutations cause an increase of the Ca^2+^-sensitivity of the myofilaments and determine an increased ATP-consumption by the cardiac sarcomeres (Ashrafian et al., 2011), thus reducing the energetic efficiency of force production by the myocardium. Both these primary sarcomeric changes are associated with an increase of [Ca^2+^]_i_: the increased myofilament Ca^2+^- sensitivity determines a slower release of Ca^2+^ from Troponin-C that in turn prolongs the decay of Ca^2+^-transients and leads to elevated diastolic [Ca^2+^]_i_ (Baudenbacher et al., 2008). ATP depletion caused by the reduced efficiency of mutated myofilaments can reduce the function of the sarcoplasmic reticulum Ca^2+^-ATPase (SERCA), thus reducing the rate of Ca^2+^ reuptake from the cytosol during relaxation (Ashrafian et al., 2003). Regardless of the cause that primarily increases [Ca^2+^]_i_, the complex remodeling of the Ca^2+^-handling apparatus in the cardiomyocytes of HCM hearts determines a global alteration of intracellular Ca^2+^ fluxes, ultimately contributing to aggravate Ca^2+^ overload in the cytosol and in the sarcoplasmic reticulum (SR) (**Figure 2**).

In human HCM cardiomyocytes, abnormalities of APD and ion current were paralleled by marked alterations of intracellular Ca^2+^ handling (Coppini et al., 2013; Ferrantini et al., 2018), as studied by virtue of fluorescence measurements using Ca^2+^-sensitive dyes. Ca^2+^ transient amplitude was similar in HCM and control myocytes. Conversely, Ca^2+^ transient kinetics was significantly slower and intracellular diastolic Ca^2+^ concentration ([Ca^2+^]_i_) was higher in HCM cells as compared to control cardiomyocytes, especially at higher rates of stimulation (**Figures 2A–C**). The elevated [Ca^2+^]_i_ contributed to the abnormally high rate of spontaneous Ca^2+^ releases from the SR observed in HCM myocytes, resulting in diastolic Ca^2+^-waves and delayed after-depolarizations (DADs), thus contributing to cellular arrhythmogenesis (**Figure 2D**). Additionally, SR Ca^2+^ content was slightly increased in HCM cardiomyocytes (**Figure 2F**), at variance with human and animal models of heart failure with reduced systolic function, where decreased SR Ca^2+^ content and Ca^2+^ transients amplitude are common observations (Beuckelmann et al., 1992; Shan et al., 2010). The altered Ca^2+^ fluxes in HCM myocytes are the result of several concurrent alterations: (i)increased amplitude and slower inactivation kinetics of L-Type Ca^2+^-current (see above), (ii)reduced expression of SERCA and reduced SERCA/phospholamban ratio (**Figure 2G**), (iii)loss or disorganization of t-tubules (see below), (iv) increase leakage of Ca^2+^ from the SR, and (v)abnormal function of the Na^+^/Ca^2+^ exchanger (NCX). The latter is the consequence of the increased intracellular concentration of Na^+^ ([Na^+^]_i_). In agreement with these results, we calculated a negative shift of the NCX reversal potential (E_NCX_), suggesting increased [Na^+^]_i_. Indeed, we directly observed an increase of [Na^+^]_i_ in HCM myocytes, as measured in human HCM myocytes using the Na^+^-selective fluorescent dye Asante Natrium Green II (**Figure 2K**). Based on this observation, we proposed that enhanced Na^+^ influx mediated by the larger I_NaL_ leads to a sustained increase in cytosolic [Na^+^]_i_, providing the driving force for an increased rate of Ca^2+^ entry trough NCX, as previously observed in secondary LV hypertrophy (Terracciano et al., 2001), albeitnot in heart failure. Combined with the increased expression of NCX and the prolongation of APs, this mechanism leads to an increased total Ca^2+^ entry during the plateau of the AP, thus helping HCM cardiomyocytes to maintain normal SR Ca^2+^ load, Ca^2+^ transients amplitude and contractility (Weisser-Thomas et al., 2003) despite SERCA down-regulation. This is at variance with reports on human failing cardiomyocytes (Beuckelmann et al., 1992), where SERCA down-regulation reduces SR Ca^2+^ load and Ca^2+^ release. However, the increased cytosolic [Na^+^]_i_ in HCM myocytes also reduces forward-mode NCX activity, thus determining the observed decrease of Ca^2+^ extrusion rate during exposure to caffeine (**Figure 2E**) and likely contributing to prolong Ca^2+^ transient decay and increase diastolic [Ca^2+^]_i_, in combination with the reduced activity of SERCA. Notably, prolongation of APs likely contributes to reduce Ca^2+^ extrusion through the NCX, as the exchanger can effectively work in forward mode only at diastolic potentials. The central role of the increased I_NaL_ in determining Na^+^ and Ca^2+^ overload in HCM cardiomyocytes is highlighted by the effects of I_NaL_ inhibition by ranolazine or GS-967 (Coppini et al., 2013; Ferrantini et al., 2018). In human HCM cardiomyocytes, besides shortening APD, I_NaL_ inhibition reduced intracellular [Na^+^] (**Figure 2K**), as directly assessed using fluorescence measurements. The reduction of [Na^+^]_i_ shifted E_NCX_ back to positive levels; this in turn led to a potentiation of the forward-mode activity of the NCX (Ca^2+^ extrusion/Na^+^ entry), while it decreased the reverse-mode function (Ca^2+^ entry/Na^+^ extrusion). The enhanced Ca^2+^ extrusion via the NCX resulted in an acceleration of Ca^2+^-transient decay, determined a reduction of diastolic [Ca^2+^]_i_, and lessened diastolic [Ca^2+^]_i_ rise in response to increases of pacing frequency (**Figures 2H–J**). In keeping with these observations, we observed that ranolazine hastened the decay of caffeine-induced Ca^2+^ transients and slightly reduced SR Ca^2+^-content in HCM cardiomyocytes (Coppini et al., 2013). Interestingly, the reduction of diastolic [Ca^2+^]_i_ by ranolazine or GS-967 reduced the occurrence of spontaneous diastolic Ca^2+^ waves, DADs and triggered activity (**Figures 2I–L**).

Intracellular Ca^2+^ overload negatively affected diastolic function in HCM myocardium: kinetics of relaxation was slower in HCM vs. control trabeculae and diastolic tension was higher, especially at high stimulation frequencies. Ranolazine and GS-967, by lowering diastolic [Ca^2+^]_i_, accelerated relaxation of HCM myocardium (Coppini et al., 2013; Ferrantini et al., 2018). Of note, when used in control myocytes and trabeculae, we observed none of the effects shown by ranolazine and GS-967 on action potentials, Ca^2+^-handling and contraction in HCM myocardium (Coppini et al., 2013; Ferrantini et al., 2018); these results highlight the selectivity of these compounds for I_NaL_ over peak I_Na_ and support the idea that I_NaL_ augmentation plays a leading role in the remodeling of cardiac electro-mechanical function in HCM.

Most of the abnormalities of Ca^2+^ handling and contraction we observed in human cardiomyocytes and trabeculae were present also in cardiomyocytes isolated from the hearts of transgenic mice carrying the clinically relevant R92Q mutation of the Troponin T gene (Coppini et al., 2017; Ferrantini et al., 2017): these include slower Ca^2+^-transients, elevated [Ca^2+^]_i_, increased I_NaL_ and [Na^+^]_i_, as well as impaired relaxation and elevated diastolic tension. Interestingly, in cardiomyocytes from the R92Q mouse, ranolazine hastened Ca^2+^ transients, normalized [Ca^2+^]_i_ and [Na^+^]_i_ and reduced Ca^2+^-dependent arrhythmias. The observed antiarrhythmic effect of ranolazine may, at least in part, depend on the direct stabilization of ryanodine receptors by the drug (Parikh et al., 2012), which may have contributed to reduce spontaneous diastolic Ca^2+^ release and arrhythmogenic Ca^2+^ waves in human and mouse HCM cardiomyocytes.

## Role of CaMKII in HCM Pathophysiology

**FIGURE 3:**
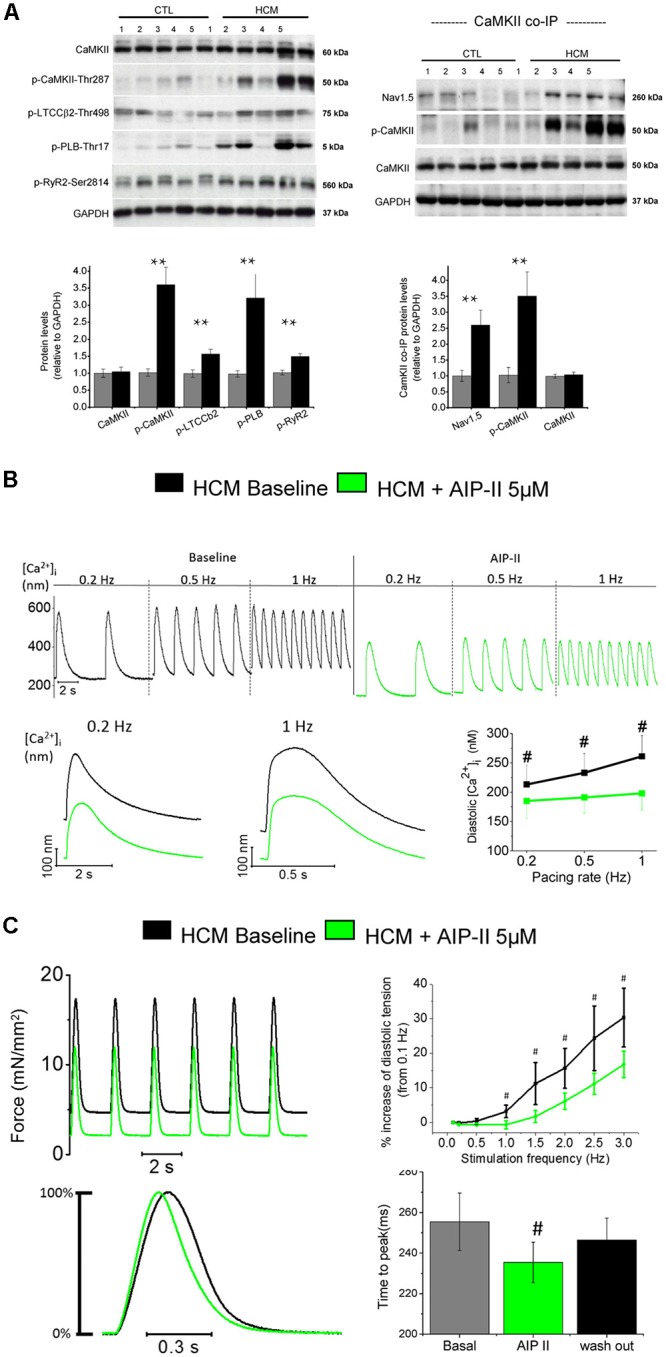
Increased CaMKII activity underlies electro-mechanical remodeling in HCM cardiomyocytes: acute effects of CaMKII block with Autocamtide 2-related inhibitory peptide II (AIP II) on Ca^2+^ transients and twitch force. **(A)** Right: Representative Western blots (above) for total CaMKII, * (phosphorylated CaMKII at threonine 287 (p-CaMKII), phosphorylated L-type Ca2^+^ channel β2 subunit at threonine 498 (p-LTCCb2), phosphorylated phospholamban at threonine 17 (p-PLB) and phosphorylated ryanodine receptor 2 at serine 2814 (p-RyR2). Average values from septum of control (*n* = 10) and HCM patients (*n* = 10) are reported below. Left: Representative Western blots (above) and mean values (below) for Co-IP of Nav1.5 with CaMKII from control (*n* = 10) and HCM patients (*n* = 10), probed with antibodies for Nav1.5, p-CaMKII and total CaMKII. **(A,B)** For each protein, 5 blots representative of the 10 are shown. Relative intensity of individual bands was quantitated and normalized to GAPDH. The ratio for control was assigned a value of 1. ^∗∗^*P* < 0.01, *t*-test. Modified from Coppini et al., 2013. **(B)** Representative Ca^2+^ transients recorded from an HCM cardiomyocytes in the absence and in the presence of AIP II, showing a reduction of diastolic [Ca^2+^**]_i_** levels upon administration of AIP II. Data from 19 cells from 4 patients **(C)** Top: Representative force traces from an HCM trabecula in the absence and in the presence of AIPII, showing a reduction of diastolic tension after AIPII and mean diastolic tension at various frequency from 6 HCM trabeculae (5 patients), in the absence and in the presence of AIP II. Bottom: Normalized representative force traces highlighting the effects of AIPII on twitch kinetics and mean data for contraction peak time, at baseline, under AIP II and after washout of the drug **(B**,**C)**. ^#^*P* < 0.05 in paired *t*-test. Previously unpublished data.)*

Sustained activation of the signaling pathways driven by calcium-calmodulin dependent protein kinase II (CaMKII) appear to play a central role as a determinant of cardiomyocyte remodeling and dysfunction in HCM myocardium (**Figure 3**), as observed in several human and animal models of cardiac hypertrophy and heart failure (Ling et al., 2009; Toischer et al., 2010; Fischer et al., 2013). Sustained activation of CaMKII in disease conditions is driven by the increase of cytosolic [Ca^2+^]_I_ within the cardiomyocyte, as Ca^2+^-bound calmodulin is the most important activator of this kinase (Hudmon and Schulman, 2002). Additionally, enhanced generation of reactive oxygen species in diseased myocytes, a likely consequence of myocardial energetic derangement, can be a strong contributor to CaMKII over-activation in HCM cardiac muscle (Erickson et al., 2011). Once activated, CaMKII then phosphorylates itself (at threonine 286 site), thereby prolonging and potentiating its activated state. In HCM vs. control specimens (Coppini et al., 2013), CaMKII auto-phosphorylation was increased 3.5-fold (**Figure 3A**), indicating increased activity (Lai et al., 1986). This in turn potentiates the phosphorylation of all the downstream targets of CaMKII (Toischer et al., 2010; Anderson et al., 2011; Fischer et al., 2013). CaMKII targets several proteins involved in the regulation of cardiomyocyte electrophysiology and calcium fluxes. The observed 2.5-fold higher phosphorylation of cardiac Na^+^ channel (NaV1.5) by CaMKII in HCM samples (**Figure 3A**; Coppini et al., 2013) may have significantly contributed to increase I_NaL_ in HCM cardiomyocytes (Wagner et al., 2006); this suggests that CaMKII activation is the most relevant cause of I_NaL_ augmentation in HCM cardiac myocytes, although additional mechanisms might be involved (e.g., oxidation of Na^+^channels) (Lu et al., 1999). The increased phosphorylation of L-Type Ca^2+^ channel (**Figure 3A**) may have contributed to slow down I_CaL_ inactivation in HCM cells (Hudmon et al., 2005; Xu et al., 2010). The slower I_CaL_ inactivation may also be a consequence of the loss of T-tubules observed in HCM cardiomyocytes (see below) (Brette et al., 2004). Moreover, the observed 1.5-fold higher CaMKII-dependent phosphorylation of RyR2 (**Figure 3A**) may have contributed to increase the rate of spontaneous releases during diastole (Shannon et al., 2003) and DADs (Curran et al., 2010) in HCM myocytes. Finally, the observed 3-fold higher phosphorylation of PLB at CaMKII site (**Figure 3A**), via reduced SERCA inhibition, may have partially counteracted the consequences of reduced SERCA expression in terms of SR Ca^2+^ reuptake, ultimately contributing to maintain SR Ca^2+^ content and steady state Ca^2+^-transient amplitude in HCM cardiomyocytes (Mattiazzi and Kranias, 2011). In support of the previous observations, we here tested the effects of the acute inhibition of CaMKII in HCM cardiomyocytes and trabeculae by using the cell-permeant version of Autocamtide-related inhibitory peptide II (AIP-II, see **Figures 3B–C**). In single human HCM cardiomyocytes, AIP-II reduced diastolic [Ca^2+^]_i_ and limited the increase of diastolic [Ca^2+^]_i_ at higher stimulation frequencies, without affecting the amplitude or the kinetics of Ca^2+^-transients (**Figure 3B**). In line with that, when used in intact human HCM while measuring twitch force, AIP-II reduced diastolic tension and limited the increase of diastolic tension upon increase of pacing rate (**Figure 3C**). Interestingly, AIP-II accelerated time-to-peak contraction in HCM trabeculae, suggesting that acute inhibition of CaMKII may directly affect the kinetics of force generation by the myofilaments.

Besides the direct effects on cardiomyocyte function, CaMKII is able to alter the expression of genes involved in the hypertrophic remodeling process of cardiomyocytes, and to facilitate the production of collagen, the increase of extracellular matrix volume and the growth of cardiac fibroblasts (Kreusser and Backs, 2014). We verified that CaMKII participates in the development and progression of cardiac functional and structural phenotype in HCM by studying the transgenic R92Q-TnT mouse model. In the R92Q-TnT mouse, CaMKII activity, increased I_NaL_ and cardiomyocyte Ca^2+^ overload go hand in hand during disease development and are present since the earliest stages of disease development (Coppini et al., 2017; Ferrantini et al., 2017). In this mouse model, lifelong treatment with ranolazine prevented the development of all features of HCM-specific cardiac phenotype, including LV thickening, progression of LV diastolic dysfunction and intra-myocardial fibrosis and the establishment of an arrhythmogenic substrate (Coppini et al., 2017). The mechanism by which ranolazine prevents the hypertrophic HCM phenotype is related to the inhibition of the enhanced I_NaL_, leading to decreased intracellular [Na^+^] and diastolic [Ca^2+^]_i_, eventually avoiding the pathological intensification of CaMKII function in treated mice (Coppini et al., 2017). The reduction of CaMKII-activity in treated mice prevented the progression of the hypertrophic remodeling in mutant hearts, thereby reducing the morphological and functional cardiac HCM-phenotype in mutation-carrier mice.

## Loss of T-Tubules in HCM Cardiomyocytes

T-tubules (Ferrantini et al., 2013) play a fundamental role in myocardial function because they allow a quick propagation of APs within the inner portions of cardiac myocytes. The simultaneous electrical activation of the whole t-tubular system allows for a synchronous triggering of Ca^2+^release from the SR across the whole myocytes, even the central regions that are farther away from surface sarcolemma. This is essential to achieve an homogeneous activation of all myofilaments and thus a rapid simultaneous shortening of the entire cardiac cell. As a proof of concept, by acutely disrupting T-tubules trough osmotic-shock, Ca^2+^release was rendered asynchronous (Brette et al., 2005), resulting in a clear impairment of contractile function and a slower relaxation (Ferrantini et al., 2014). In cardiomyocytes from animal models of cardiac hypertrophy and heart failure, a delay of local Ca^2+^release was observed both in areas where t-tubules are disrupted (Song et al., 2006) and in regions adjacent to electrically uncoupled T-tubules (Sacconi et al., 2012; Crocini et al., 2014). Lyon et al. (2009) studied T-tubular structures in myocardial specimens from patients with HF caused by different diseases (i.e., post-ischemic HF, dilated cardiomyopathy and HCM) and observed a significant reduction of T-tubule density in all failing human hearts regardless of the underlying disease, including in end-stage HCM. In a mouse model of HCM we used Random Access Multi Photon (RAMP) microscopy to measure the local propagation of APs in the T-tubule and the correspondent release of Ca^2+^in the adjacent junctional area by simultaneously mapping multiple sites of an isolated cell. With this technique, we found that more than 20% of T-tubules are unable to propagate AP and the surrounding junctional regions display a significantly delayed local Ca^2+^release (Crocini et al., 2016). In this mouse model, asynchronous intracellular Ca^2+^ release due to altered T-tubules contribute to slow down Ca^2+^-transient kinetics and impair diastolic function.

**FIGURE 4:**
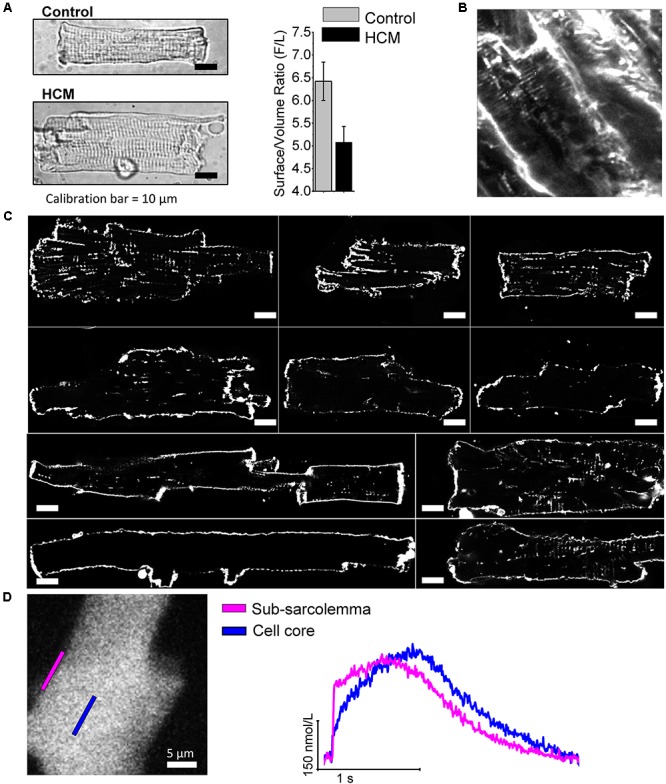
The density of T-tubules is markedly low in HCM cardiomyocytes. **(A)** Left: Representative images of a control (top) and an HCM(bottom) cardiomyocyte, showing cell hypertrophy in HCM. Right: surface/volume ratio in HCM and control cardiomyocytes;surface is derived from cell capacitance, volume estimated from cell area. Data from 64 cells (14 patients). Previously unpublished data. **(B)** 2-photon image recorded in HCM intact tissue after membrane labeling with anepp dyes, showing a severe reduction of T-tubules. **(C)**Each cell derives from a different patient sample (ID of the patient is indicated next to the cell in each respective image). Cells were stained with Di-3-ANEPPDHQ (Thermo-Fisher) and imaged with a Leica Confocal microscope using the 488 nm laser line. Sections were taken at mid cell. While the outer sarcolemma is well stained in all myocytes, T-tubules are barely visible in most of them and some cells are completely devoid of T-tubules. White bars equal 10 μm. **(D)** Recordings of intracellular calcium from an HCM myocytes using a fast camera; right: calcium variations during an elicited electrical activation in the subsarcolemma and in the cell core. In agreement with the loss of t-tubule, calcium rise in the core is significantly delayed. Previously unpublished data.

In all cardiomyocytes subjected to patch-clamp measurements, we measured cell capacitance, an index of sarcolemma extension, and compared with cell volume, as estimated by cell surface (**Figure 4A**). The ratio between cell capacitance and volume (surface/volume ratio) was reduced in HCM vs. control human cardiomyocytes, suggesting reduction of T-tubules. In addition, we recently performed a preliminary assessment of the density of T-tubules in cardiomyocytes isolated from surgical septal samples of 10 HCM patient (Ferrantini et al., 2018; **Figures 4B,C**): in all the myocytes studied under the confocal microscope after membrane fluorescent labeling, we observed a very low density of T-tubules, much lower than what is expected in healthy human cardiomyocytes (Lyon et al., 2009). We previously demonstrated that the loss of t-tubules may directly contribute to slow down the kinetics of Ca^2+^ transients (Ferrantini et al., 2014), thus delaying relaxation, with possible detrimental effects on diastolic function. However, it is unclear whether and how loss of t-tubules affects the propensity toward arrhythmias of HCM cardiomyocytes. Loss of t-tubules could be protective against arrhythmias because orphan RyR channels (RyR uncoupled from t-tubules) have a reduced likelihood of diastolic spontaneous opening (Brette et al., 2005); however, the rate of Ca^2+^ waves is increased in human HCM. Loss of t-tubules leads to reduction of capacitance/volume ratio, thus increasing conduction velocity in detubulated myocardial tissue: this effect may prevent the formation of small re-entry circuits. On the contrary, loss of t-tubules may promote arrhythmias because it reduces synchronicity of Ca^2+^ release, thus raising the likelihood of APD and effective-refractory-period (ERP) temporal fluctuations (alternans): APD and ERP alternans facilitate the formation of dynamic reentry circuits (Heinzel et al., 2011). In support of this, we found that Ca^2+^ release is indeed asynchronous in human HCM cardiomyocytes (**Figure 4D**). Moreover, loss of t-tubules reduces Ca^2+^-dependent inactivation of I_CaL_, as subsarcolemmal systolic [Ca^2+^] is lower below surface sarcolemma than in proximity of T-tubules (Morotti et al., 2012): indeed, I_CaL_ inactivation is slower in human HCM cells as compared with controls. Finally, the presence of dysfunctional residual t-tubules within the myocyte may facilitate spontaneous Ca^2+^-release events at SR sites adjacent to those tubules, as we previously showed in a model of heart failure (Crocini et al., 2014), increasing the overall risk of Ca^2+^ waves and DADs.

## Abnormal Response of HCM Myocardium to Beta-Adrenergic Stimulation

**FIGURE 5:**
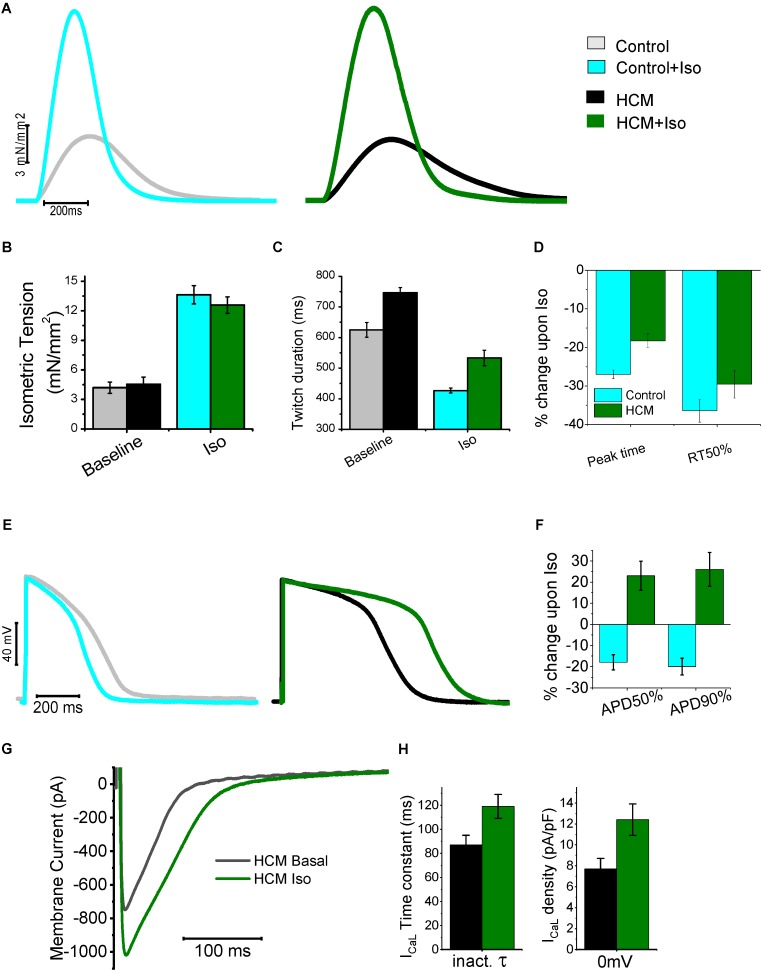
Mechanical and electrical response to β-adrenergic stimulation of HCM myocardium. **(A)** Representative superimposed force twitches elicited at 0.5 Hz in control (left) and HCM (right) trabeculae in the absence and presence of Isoproterenol 10^-7^M (Iso). **(B)** Isometric tension during steady state stimulation at 0.5 in the absence and presence of Iso in control and HCM trabeculae. **(C)** Duration of force twitches (from stimulus to 90% of relaxation) elicited at 0.5 Hz in the absence and presence of Isoproterenol 10^-7^M. **(D)** Percentages of Change in the parameters of twitch kinetics (0.5 Hz) upon exposure to Iso in Control (cyan) and HCM (green) trabeculae: time from stimulus to peak contraction (peak time) and time from peak to 50% of relaxation (RT50%). **(E)** Representative superimposed action potentials elicited at 0.5 Hz in control (left) and HCM (right) cardiomyocytes, in the absence and presence of Iso. **(F)** Percentages of Change in the parameters of action potential kinetics upon exposure to Iso in Control (cyan) and HCM (green) cardiomyocytes: time from stimulus to 50% repolarization (APD50%) and time from peak to 90% of repolarization (APD90%). **(G)** Representative superimposed L-Type Ca-current traces at baseline (black traces) and in the presence of Iso (green). **(H)** L-Type Ca-current inactivation time-constant(left) and density (right) at baseline (black) and with Iso (green) in HCM cells. Modified from Ferrantini et al. (2018).

We recently investigated the characteristics and anomalies of β-adrenergic signaling in the myocardium of HCM patients by comparing the response of control cardiac muscle to β-adrenergic stimulation with that observed in HCM cardiomyocytes or trabeculae (Ferrantini et al., 2018; **Figure 5**). In particular, we observed that the mechanical response to the β-adrenergic agonist isoproterenol (that is, augmentation of twitch force amplitude and acceleration of relaxation) was qualitatively similar in HCM and control trabeculae, albeit the kinetics of contraction and relaxation remained slower in HCM myocardium, even upon maximal β-adrenergic activation (Ferrantini et al., 2018; **Figures 5A–D**). These results suggest that the molecular mechanisms responsible for the acceleration of relaxation upon β-adrenergic activation [i.e., myofilament Ca2^+^-desensitization (Sequeira et al., 2013) and phospholamban phosphorylation (Coppini et al., 2013; Helms et al., 2016)] are preserved in HCM myocardium.

On the contrary The electrical response to isoprenalin observed in single HCM cardiomyocytes was profoundly different as compared with control cardiac cells (**Figures 5E–G**). Activation of β-adrenergic receptors physiologically leads to the enhancement of both I_CaL_ and the slow delayed-rectifier K^+^ current (I_Ks_). In healthy human cardiomyocytes the increase of repolarizing K^+^ currents prevails over the augmentation of I_CaL_ (Terrenoire et al., 2005), thus determining a net reduction of AP duration (Taggart et al., 2003; **Figure 5E**). In HCM cardiomyocytes, however, we observed unbalanced changes in the expression of Ca^2+^ and K^+^ currents (Coppini et al., 2013). As shown in **Figure 1**, we observed a reduction of the expression of all K^+^ channels (including I_Ks_) while the expression of Ca^2+^ channels and the density of I_CaL_ was slightly increased. Therefore, in response to β-stimulation, the potentiation of I_CaL_ prevails over the increase of K^+^ currents, ultimately causing a net increase of inward currents during the plateau of the AP, thus determining a “paradoxical” prolongation of APD in HCM cardiomyocytes (Ferrantini et al., 2018; **Figures 5E,F**). Moreover, we observed that β-adrenergic stimulation in HCM cardiomyocytes not only increased peak I_CaL_ amplitude, but also slowed down I_CaL_ inactivation (Ferrantini et al., 2018; **Figures 5G,H**), further contribute to the prolongation of APs by β-adrenergic agonists. Additionally, recent work suggested that β-adrenergic stimulation is able to rapidly and transiently increase I_NaL_ in cardiac myocytes (Dybkova et al., 2014): a further augmentation of the already increased I_NaL_ in HCM myocytes may have played a relevant role in the paradoxical prolongation of APs with isoprenaline. As expected, AP prolongation by β-adrenergic agonists further increased the occurrence of arrhythmogenic early afterdepolarizations, triggered activity and spontaneous premature contractions in HCM cardiomyocytes and trabeculae (Ferrantini et al., 2018). This paradoxical electrical response may have severe consequences in HCM patients, that is, it may increase the risk of exercise/stress-induced arrhythmias in HCM patients.

In parallel, the response of HCM cardiomyocytes to β-adrenergic stimulation in terms of Ca^2+^-handling changes was also abnormal. In HCM cardiomyocytes the β-adrenergic-induced increase of Ca^2+^ release may primarily rely on the prolongation of Ca^2+^ entry via I_CaL_ channels caused by the slower current inactivation. In addition to the increase of net Ca^2+^ influx through I_CaL_, the potentiation of Ca^2+^-entry via reverse-mode NCX may have contributed to the augmentation of Ca^2+^-transients β-receptor activation (Perchenet et al., 2000). The increase of NCX-mediated Ca^2+^-entry upon β-adrenergic stimulation is probably caused by the rise of intracellular [Na^+^] in response to the transient augmentation of I_NaL_ and by the further prolongation of AP plateau, as reverse-mode NCX is only active at positive membrane potentials (Coppini et al., 2013; Dybkova et al., 2014). The idea that the increase of contractile tension in response to β-adrenergic activation mainly depends on the larger Ca^2+^-entry through the sarcolemma apparently contrasts with the largely accepted idea that the inotropic response of β-stimulus mainly stems from the increase of SR Ca^2+^ content (Desantiago et al., 2008), mediated by the enhancement of SR Ca^2+^-uptake by SERCA via PKA-dependent phospholamban phosphorylation, ultimately causing an enhanced release of Ca^2+^ from the SR. The hastening of Ca-transient decay in response to isoprenaline in HCM cardiomyocytes (Ferrantini et al., 2018) suggests that the β-adrenergic-induced increase of SERCA function (via PkA-mediated phospholamban phosphorylation) is preserved in HCM myocardium, as Ca-transient decay is physiologically accelerated by isoproterenol. Despite the shortening of Ca^2+^ transient decay, their rise-time is so prolonged that the total duration of Ca^2+^-transients is not reduced upon β-stimulation. The rise of SR Ca^2+^ load upon exposure to isoprenaline in HCM myocytes may be limited by the increased phosphorylation of ryanodine receptors (due to the higher CaMKII activity), which in turn causes a larger rate of Ca^2+^ leakage from the SR during the diastolic period. This may render positive inotropic responses more dependent on the increase of Ca^2+^ entry from the sarcolemma, as the possibility to accumulate Ca^2+^ in the SR is limited. An additional contributor to this aberrant behavior is the lower density of t-tubules observed in HCM myocytes (see above) (Orchard and Brette, 2008). The physiological response to β-adrenergic activation may be radically different in disease-remodeled myocytes showing a sparse and disorganized the T-tubular system. In HCM cells, where T-tubules are nearly absent, we expect a large redistribution of I_CaL_ channels to the surface sarcolemma (Coppini et al., 2013). Under such conditions, modulation of cellularinotropism essentially relies on the magnitude of sarcolemmal Ca^2+^ triggers (Ferrantini et al., 2014), that is, the amplitude and duration of I_CaL_ plus the rate of NCX-mediated Ca^2+^ entry (reverse mode). The prolongation of APs causing increased sarcolemmal Ca^2+^-entry appears to be an essential requisite for the preservation of the positive-inotropic effect of β-adrenergic stimulation in HCM myocardium. In line with that hypothesis, the application of ranolazine or GS-967 on top of isoproterenol, which prevented the β-adrenergic-induced AP prolongation, also greatly reduced the positive inotropic response to β-stimulation in HCM myocardium (Ferrantini et al., 2018). The preservation of inotropic response in HCM myocardium comes at the expense of a further impairment of diastolic function (due to prolonged Ca^2+^ transient rise-time) and a further increase of the likelihood of cellular arrhythmias (due to increased cytosolic Ca^2+^ accumulation and AP prolongation).

## Conclusion

**FIGURE 6:**
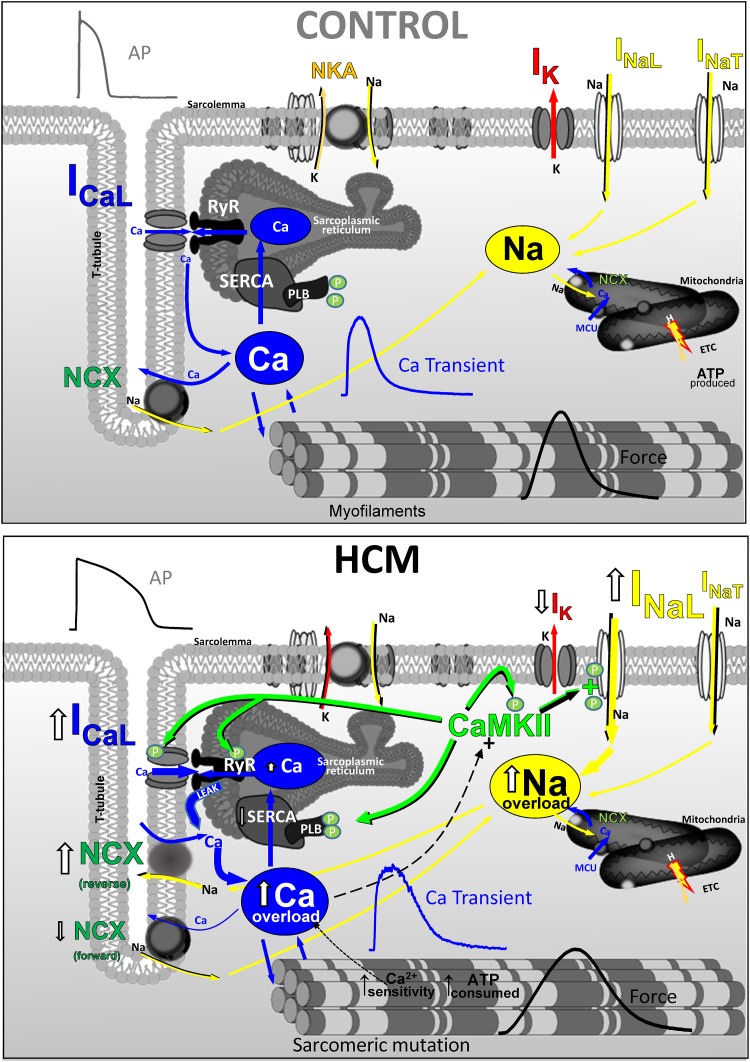
Functional changes of ion currernts and EC-coupling in human HCM vs. control myocardium (CARTOON).

Hypertrophic remodeling in the myocardium of patients with HCM features several pathological alterations of cardiomyocyte electrical function and intracellular Ca^2+^-handling (**Figure 6**), that contribute to increase the likelihood of EADs, DADs and premature contractions. Such arrhythmogenic changes occur since the early stages of HCM disease development and may explain the occurrence of ventricular arrhythmias and cardiac arrest in young HCM patients who lack advanced structural alterations of myocardial structure (e.g., diffuse fibrosis, myocardial scars, massive hypertrophy, severe microvascular abnormalities). Changes of the expression and function of ion channels and EC-coupling proteins in HCM myocardium concur to generate an “acquired channelopathy” phenotype in HCM patients that raises the risk of arrhythmias even before the establishment of a structural substrate for sustained arrhythmias. Among the several molecular determinants of cellular arrrhythmogenesis, increased I_NaL_ appear to play a leading role in HCM and may represent a selective target for pharmacological prevention of arrhythmias in this disease, with possible clinical implications. Although targeting I_NaL_ is proven to be effective antiarrhytmic approach, one cannot discount NCX (Passini et al., 2016), leaky RyR channels or cardiac CaMKII as potential targets to reduce Ca^2+^-dependent arrhythmias in HCM.

## Author Contributions

RC performed most of the original experiments described in this review and drafted this manuscript. CF contributed to the original data presented and discussed here, drafted the figures and copyedited the manuscript. AM, CP, and EC supervised the original projects that led to production of the results shown here; moreover, they critically reviewed and edited the manuscript. All authors concur with the current submitted version.

## Conflict of Interest Statement

The authors declare that the research was conducted in the absence of any commercial or financial relationships that could be construed as a potential conflict of interest.

## References

[B1] Adabag A. S. Casey S. A. Kuskowski M. A. Zenovich A. G. Maron B. J. (2005). Spectrum and prognostic significance of arrhythmias on ambulatory Holter electrocardiogram in hypertrophic cardiomyopathy. *J. Am. Coll. Cardiol.* 45 697–704. 10.1016/j.jacc.2004.11.043 15734613

[B2] Anderson M. E. Brown J. H. Bers D. M. (2011). CaMKII in myocardial hypertrophy and heart failure. *J. Mol. Cell Cardiol.* 51 468–473. 10.1016/j.yjmcc.2011.01.012 21276796 PMC3158288

[B3] Antzelevitch C. Belardinelli L. (2006). The role of sodium channel current in modulating transmural dispersion of repolarization and arrhythmogenesis. *J. Cardiovasc. Electrophysiol.* 17(Suppl. 1), S79–S85. 10.1111/j.1540-8167.2006.00388.x 16686686 PMC1474079

[B4] Antzelevitch C. Belardinelli L. Zygmunt A. C. Burashnikov A. Di Diego J. M. Fish J. M. (2004). Electrophysiological effects of ranolazine, a novel antianginal agent with antiarrhythmic properties. *Circulation* 110 904–910. 10.1161/01.CIR.0000139333.83620.5D 15302796 PMC1513623

[B5] Ashrafian H. McKenna W. J. Watkins H. (2011). Disease pathways and novel therapeutic targets in hypertrophic cardiomyopathy. *Circ. Res.* 109 86–96. 10.1161/CIRCRESAHA.111.242974 21700950

[B6] Ashrafian H. Redwood C. Blair E. Watkins H. (2003). Hypertrophic cardiomyopathy:a paradigm for myocardial energy depletion. *Trends Genet.* 19 263–268. 10.1016/S0168-9525(03)00081-87 12711218

[B7] Authors/Task Force Members Elliott P. M. Anastasakis A. Borger M. A. Borggrefe M. Cecchi F. (2014). 2014 ESC Guidelines on diagnosis and management of hypertrophic cardiomyopathy: the Task Force for the Diagnosis and Management of Hypertrophic Cardiomyopathy of the European Society of Cardiology (ESC). *Eur. Heart J.* 35 2733–2779. 10.1093/eurheartj/ehu284 25173338

[B8] Avanesov M. Munch J. Weinrich J. Well L. Saring D. Stehning C. (2017). Prediction of the estimated 5-year risk of sudden cardiac death and syncope or non-sustained ventricular tachycardia in patients with hypertrophic cardiomyopathy using late gadolinium enhancement and extracellular volume CMR. *Eur. Radiol.* 27 5136–5145. 10.1007/s00330-017-4869-x 28616729

[B9] Baudenbacher F. Schober T. Pinto J. R. Sidorov V. Y. Hilliard F. Solaro R. J. (2008). Myofilament Ca^2+^ sensitization causes susceptibility to cardiac arrhythmia in mice. *J. Clin. Invest.* 118 3893–3903. 10.1172/JCI36642 19033660 PMC2582931

[B10] Beuckelmann D. J. Nabauer M. Erdmann E. (1992). Intracellular calcium handling in isolated ventricular myocytes from patients with terminal heart failure. *Circulation* 85 1046–1055. 10.1161/01.CIR.85.3.1046 1311223

[B11] Brette F. Despa S. Bers D. M. Orchard C. H. (2005). Spatiotemporal characteristics of SR Ca^2+^ uptake and release in detubulated rat ventricular myocytes. *J. Mol. Cell Cardiol.* 39 804–812. 10.1016/j.yjmcc.2005.08.005 16198369

[B12] Brette F. Salle L. Orchard C. H. (2004). Differential modulation of L-type Ca2 + current by SR Ca^2+^ release at the T-tubules and surface membrane of rat ventricular myocytes. *Circ. Res.* 95 e1–e7. 10.1161/01.RES.0000135547.53927.F6 15192026

[B13] Cecchi F. Olivotto I. Gistri R. Lorenzoni R. Chiriatti G. Camici P. G. (2003). Coronary microvascular dysfunction and prognosis in hypertrophic cardiomyopathy. *N. Engl. J. Med.* 349 1027–1035. 10.1056/NEJMoa025050 12968086

[B14] Chan R. H. Maron B. J. Olivotto I. Pencina M. J. Assenza G. E. Haas T. (2014). Prognostic value of quantitative contrast-enhanced cardiovascular magnetic resonance for the evaluation of sudden death risk in patients with hypertrophic cardiomyopathy. *Circulation* 130 484–495. 10.1161/CIRCULATIONAHA.113.007094 25092278

[B15] Cho J. H. Zhang R. Kilfoil P. J. Gallet R. de Couto G. Bresee C. (2017). Delayed repolarization underlies ventricular arrhythmias in rats with heart failure and preserved ejection fraction. *Circulation* 136 2037–2050. 10.1161/CIRCULATIONAHA.117.028202 28974519 PMC5698112

[B16] Coppini R. Ferrantini C. Yao L. Fan P. Del Lungo M. Stillitano F. (2013). Late sodium current inhibition reverses electromechanical dysfunction in human hypertrophic cardiomyopathy. *Circulation* 127 575–584. 10.1161/CIRCULATIONAHA.112.134932 23271797

[B17] Coppini R. Mazzoni L. Ferrantini C. Gentile F. Pioner J. M. Laurino A. (2017). Ranolazine prevents phenotype development in a mouse model of hypertrophic cardiomyopathy. *Circ. Heart Fail.* 10:e003565. 10.1161/CIRCHEARTFAILURE.116.003565 28255011 PMC6284403

[B18] Crocini C. Coppini R. Ferrantini C. Yan P. Loew L. M. Tesi C. (2014). Defects in T-tubular electrical activity underlie local alterations of calcium release in heart failure. *Proc. Natl. Acad. Sci. U.S.A.* 111 15196–15201. 10.1073/pnas.1411557111 25288764 PMC4210349

[B19] Crocini C. Ferrantini C. Scardigli M. Coppini R. Mazzoni L. Lazzeri E. (2016). Novel insights on the relationship between T-tubular defects and contractile dysfunction in a mouse model of hypertrophic cardiomyopathy. *J. Mol. Cell Cardiol.* 91 42–51. 10.1016/j.yjmcc.2015.12.013 26714042 PMC4767219

[B20] Curran J. Brown K. H. Santiago D. J. Pogwizd S. Bers D. M. Shannon T. R. (2010). Spontaneous Ca waves in ventricular myocytes from failing hearts depend on Ca^2+^-calmodulin-dependent protein kinase II. *J. Mol. Cell Cardiol.* 49 25–32. 10.1016/j.yjmcc.2010.03.013 20353795 PMC2883657

[B21] Dass S. Suttie J. J. Piechnik S. K. Ferreira V. M. Holloway C. J. Banerjee R. (2012). Myocardial tissue characterization using magnetic resonance noncontrast t1 mapping in hypertrophic and dilated cardiomyopathy. *Circ. Cardiovasc. Imaging* 5 726–733. 10.1161/CIRCIMAGING.112.976738 23071146

[B22] Desantiago J. Ai X. Islam M. Acuna G. Ziolo M. T. Bers D. M. (2008). Arrhythmogenic effects of beta2-adrenergic stimulation in the failing heart are attributable to enhanced sarcoplasmic reticulum Ca load. *Circ. Res.* 102 1389–1397. 10.1161/CIRCRESAHA.107.169011 18467626 PMC2585979

[B23] Dybkova N. Wagner S. Backs J. Hund T. J. Mohler P. J. Sowa T. (2014). Tubulin polymerization disrupts cardiac beta-adrenergic regulation of late INa. *Cardiovasc. Res.* 103 168–177. 10.1093/cvr/cvu120 24812278 PMC4133594

[B24] Erickson J. R. He B. J. Grumbach I. M. Anderson M. E. (2011). CaMKII in the cardiovascular system: sensing redox states. *Physiol. Rev.* 91 889–915. 10.1152/physrev.00018.2010 21742790 PMC3732780

[B25] Ferrantini C. Coppini R. Pioner J. M. Gentile F. Tosi B. Mazzoni L. (2017). Pathogenesis of hypertrophic cardiomyopathy is mutation rather than disease specific: a comparison of the cardiac troponin T E163R and R92Q mouse models. *J. Am. Heart Assoc.* 6 e005407. 10.1161/JAHA.116.005407 28735292 PMC5586279

[B26] Ferrantini C. Coppini R. Sacconi L. Tosi B. Zhang M. L. Wang G. L. (2014). Impact of detubulation on force and kinetics of cardiac muscle contraction. *J. Gen. Physiol.* 143 783–797. 10.1085/jgp.201311125 24863933 PMC4035744

[B27] Ferrantini C. Crocini C. Coppini R. Vanzi F. Tesi C. Cerbai E. (2013). The transverse-axial tubular system of cardiomyocytes. *Cell Mol. Life Sci.* 70 4695–4710. 10.1007/s00018-013-1410-1415 23846763 PMC11113601

[B28] Ferrantini C. Pioner J. M. Mazzoni L. Gentile F. Tosi B. Rossi A. (2018). Late sodium current inhibitors to treat exercise-induced obstruction in hypertrophic cardiomyopathy: an in vitro study in human myocardium. *Br. J. Pharmacol.* 175 2635–2652. 10.1111/bph.14223 29579779 PMC6003658

[B29] Fischer T. H. Herting J. Tirilomis T. Renner A. Neef S. Toischer K. (2013). Ca2 + /calmodulin-dependent protein kinase II and protein kinase A differentially regulate sarcoplasmic reticulum Ca2 + leak in human cardiac pathology. *Circulation* 128 970–981. 10.1161/CIRCULATIONAHA.113.001746 23877259

[B30] Fraysse B. Weinberger F. Bardswell S. C. Cuello F. Vignier N. Geertz B. (2012). Increased myofilament Ca^2+^ sensitivity and diastolic dysfunction as early consequences of Mybpc3 mutation in heterozygous knock-in mice. *J. Mol. Cell Cardiol.* 52 1299–1307. 10.1016/j.yjmcc.2012.03.009 22465693 PMC3370652

[B31] Galati G. Leone O. Pasquale F. Olivotto I. Biagini E. Grigioni F. (2016). Histological and histometric characterization of myocardial fibrosis in end-stage hypertrophic cardiomyopathy: a clinical-pathological study of 30 explanted hearts. *Circ. Heart Fail.* 9:e003090. 10.1161/CIRCHEARTFAILURE.116.003090 27618852

[B32] Gersh B. J. Maron B. J. Bonow R. O. Dearani J. A. Fifer M. A. Link M. S. (2011). 2011 ACCF/AHA guideline for the diagnosis and treatment of hypertrophic cardiomyopathy: a report of the American College of Cardiology Foundation/American Heart Association Task Force on Practice Guidelines. *Circulation* 124 e783–e831. 10.1161/CIR.0b013e318223e2bd 22068434

[B33] Grandi E. Pasqualini F. S. Bers D. M. (2010). A novel computational model of the human ventricular action potential and Ca transient. *J. Mol. Cell Cardiol.* 48 112–121. 10.1016/j.yjmcc.2009.09.019 19835882 PMC2813400

[B34] Haim T. E. Dowell C. Diamanti T. Scheuer J. Tardiff J. C. (2007). Independent FHC-related cardiac troponin T mutations exhibit specific alterations in myocellular contractility and calcium kinetics. *J. Mol. Cell Cardiol.* 42 1098–1110. 10.1016/j.yjmcc.2007.03.906 17490679

[B35] Heinzel F. R. MacQuaide N. Biesmans L. Sipido K. (2011). Dyssynchrony of Ca^2+^ release from the sarcoplasmic reticulum as subcellular mechanism of cardiac contractile dysfunction. *J. Mol. Cell Cardiol.* 50 390–400. 10.1016/j.yjmcc.2010.11.008 21075114

[B36] Helms A. S. Alvarado F. J. Yob J. Tang V. T. Pagani F. Russell M. W. (2016). Genotype-dependent and -independent calcium signaling dysregulation in human hypertrophic cardiomyopathy. *Circulation* 134 1738–1748. 10.1161/CIRCULATIONAHA.115.020086 27688314 PMC5127749

[B37] Ho C. Y. Abbasi S. A. Neilan T. G. Shah R. V. Chen Y. Heydari B. (2013). T1 measurements identify extracellular volume expansion in hypertrophic cardiomyopathy sarcomere mutation carriers with and without left ventricular hypertrophy. *Circ. Cardiovasc. Imaging* 6 415–422. 10.1161/CIRCIMAGING.112.000333 23549607 PMC3769196

[B38] Hudmon A. Schulman H. (2002). Structure-function of the multifunctional Ca2 + /calmodulin-dependent protein kinase II. *Biochem. J.* 364(Pt 3) 593–611. 10.1042/BJ20020228 11931644 PMC1222606

[B39] Hudmon A. Schulman H. Kim J. Maltez J. M. Tsien R. W. Pitt G. S. (2005). CaMKII tethers to L-type Ca^2+^ channels, establishing a local and dedicated integrator of Ca^2+^ signals for facilitation. *J. Cell Biol.* 171 537–547. 10.1083/jcb.200505155 16275756 PMC1343528

[B40] Hurtado-de-Mendoza D. Corona-Villalobos C. P. Pozios I. Gonzales J. Soleimanifard Y. Sivalokanathan S. (2017). Diffuse interstitial fibrosis assessed by cardiac magnetic resonance is associated with dispersion of ventricular repolarization in patients with hypertrophic cardiomyopathy. *J. Arrhythm* 33 201–207. 10.1016/j.joa.2016.10.005 28607615 PMC5459419

[B41] Johnson J. N. Grifoni C. Bos J. M. Saber-Ayad M. Ommen S. R. Nistri S. (2011). Prevalence and clinical correlates of QT prolongation in patients with hypertrophic cardiomyopathy. *Eur. Heart J.* 32 1114–1120. 10.1093/eurheartj/ehr021 21345853 PMC3086898

[B42] Knollmann B. C. Kirchhof P. Sirenko S. G. Degen H. Greene A. E. Schober T. (2003). Familial hypertrophic cardiomyopathy-linked mutant troponin T causes stress-induced ventricular tachycardia and Ca^2+^-dependent action potential remodeling. *Circ. Res.* 92 428–436. 10.1161/01.RES.0000059562.91384.1A 12600890

[B43] Kreusser M. M. Backs J. (2014). Integrated mechanisms of CaMKII-dependent ventricular remodeling. *Front. Pharmacol.* 5:36. 10.3389/fphar.2014.00036 24659967 PMC3950490

[B44] Lai Y. Nairn A. C. Greengard P. (1986). Autophosphorylation reversibly regulates the Ca^2+^/calmodulin-dependence of Ca^2+^/calmodulin-dependent protein kinase II. *Proc. Natl. Acad. Sci. U.S.A.* 83 4253–4257. 10.1073/pnas.83.12.4253 3012560 PMC323710

[B45] Lan F. Lee A. S. Liang P. Sanchez-Freire V. Nguyen P. K. Wang L. (2013). Abnormal calcium handling properties underlie familial hypertrophic cardiomyopathy pathology in patient-specific induced pluripotent stem cells. *Cell Stem Cell* 12 101–113. 10.1016/j.stem.2012.10.010 23290139 PMC3638033

[B46] Ling H. Zhang T. Pereira L. Means C. K. Cheng H. Gu Y. (2009). Requirement for Ca^2+^/calmodulin-dependent kinase II in the transition from pressure overload-induced cardiac hypertrophy to heart failure in mice. *J. Clin. Invest.* 119 1230–1240. 10.1172/JCI38022 19381018 PMC2673879

[B47] Lu T. Lee H. C. Kabat J. A. Shibata E. F. (1999). Modulation of rat cardiac sodium channel by the stimulatory G protein alpha subunit. *J. Physiol.* 518(Pt 2) 371–384. 10381586 10.1111/j.1469-7793.1999.0371p.x PMC2269432

[B48] Lyon A. R. MacLeod K. T. Zhang Y. Garcia E. Kanda G. K. Lab M. J. (2009). Loss of T-tubules and other changes to surface topography in ventricular myocytes from failing human and rat heart. *Proc. Natl. Acad. Sci. U.S.A.* 106 6854–6859. 10.1073/pnas.0809777106 19342485 PMC2672472

[B49] Maltsev V. A. Sabbah H. N. Higgins R. S. Silverman N. Lesch M. Undrovinas A. I. (1998). Novel, ultraslow inactivating sodium current in human ventricular cardiomyocytes. *Circulation* 98 2545–2552. 10.1161/01.CIR.98.23.2545 9843461

[B50] Maron B. J. McKenna W. J. Danielson G. K. Kappenberger L. J. Kuhn H. J. Seidman C. E. (2003). American College of Cardiology/European Society of Cardiology clinical expert consensus document on hypertrophic cardiomyopathy. A report of the American College of Cardiology Foundation Task Force on Clinical Expert Consensus Documents and the European Society of Cardiology Committee for Practice Guidelines. *J. Am. Coll. Cardiol.* 42 1687–1713. 10.1016/S0735-1097(03)00941-0 14607462

[B51] Maron B. J. Olivotto I. Spirito P. Casey S. A. Bellone P. Gohman T. E. (2000). Epidemiology of hypertrophic cardiomyopathy-related death: revisited in a large non-referral-based patient population. *Circulation* 102 858–864. 10.1161/01.CIR.102.8.858 10952953

[B52] Maron M. S. Rowin E. J. Olivotto I. Casey S. A. Arretini A. Tomberli B. (2016). Contemporary natural history and management of nonobstructive hypertrophic cardiomyopathy. *J. Am. Coll. Cardiol.* 67 1399–1409. 10.1016/j.jacc.2016.01.023 27012399

[B53] Mattiazzi A. Kranias E. G. (2011). CaMKII regulation of phospholamban and SR Ca^2+^ load. *Heart Rhythm* 8 784–787. 10.1016/j.hrthm.2010.11.035 21111063 PMC3081991

[B54] Maurizi N. Passantino S. Spaziani G. Girolami F. Arretini A. Targetti M. (2018). Long-term outcomes of pediatric-onset hypertrophic cardiomyopathy and age-specific risk factors for lethal arrhythmic events. *JAMA Cardiol.* 3 520–525. 10.1001/jamacardio.2018.0789 29710196 PMC6128509

[B55] Morotti S. Grandi E. Summa A. Ginsburg K. S. Bers D. M. (2012). Theoretical study of L-type Ca^2+^ current inactivation kinetics during action potential repolarization and early afterdepolarizations. *J. Physiol.* 590 4465–4481. 10.1113/jphysiol.2012.231886 22586219 PMC3477752

[B56] Olivotto I. Cecchi F. Poggesi C. Yacoub M. H. (2012). Patterns of disease progression in hypertrophic cardiomyopathy: an individualized approach to clinical staging. *Circ. Heart Fail.* 5 535–546. 10.1161/CIRCHEARTFAILURE.112.967026 22811549

[B57] Olivotto I. Girolami F. Nistri S. Rossi A. Rega L. Garbini F. (2009). The many faces of hypertrophic cardiomyopathy: from developmental biology to clinical practice. *J. Cardiovasc. Transl. Res.* 2 349–367. 10.1007/s12265-009-9137-9132 20559994

[B58] Orchard C. Brette F. (2008). t-Tubules and sarcoplasmic reticulum function in cardiac ventricular myocytes. *Cardiovasc. Res.* 77 237–244. 10.1093/cvr/cvm002 18006490

[B59] Parikh A. Mantravadi R. Kozhevnikov D. Roche M. A. Ye Y. Owen L. J. (2012). Ranolazine stabilizes cardiac ryanodine receptors: a novel mechanism for the suppression of early afterdepolarization and torsades de pointes in long QT type 2. *Heart Rhythm* 9 953–960. 10.1016/j.hrthm.2012.01.010 22245792 PMC3335957

[B60] Passini E. Minchole A. Coppini R. Cerbai E. Rodriguez B. Severi S. (2016). Mechanisms of pro-arrhythmic abnormalities in ventricular repolarisation and anti-arrhythmic therapies in human hypertrophic cardiomyopathy. *J. Mol. Cell Cardiol.* 96 72–81. 10.1016/j.yjmcc.2015.09.003 26385634 PMC4915817

[B61] Perchenet L. Hinde A. K. Patel K. C. Hancox J. C. Levi A. J. (2000). Stimulation of Na/Ca exchange by the beta-adrenergic/protein kinase A pathway in guinea-pig ventricular myocytes at 37 degrees C. *Pflugers Arch.* 439 822–828. 10784358 10.1007/s004249900218

[B62] Pieske B. Houser S. R. (2003). [Na + ]i handling in the failing human heart. *Cardiovasc. Res.* 57 874–886. 10.1016/S0008-6363(02)00841-6 12650866

[B63] Pogwizd S. M. Corr P. B. (1987). Electrophysiologic mechanisms underlying arrhythmias due to reperfusion of ischemic myocardium. *Circulation* 76 404–426. 10.1161/01.CIR.76.2.404 3608126

[B64] Pogwizd S. M. Sipido K. R. Verdonck F. Bers D. M. (2003). Intracellular Na in animal models of hypertrophy and heart failure: contractile function and arrhythmogenesis. *Cardiovasc. Res.* 57 887–896. 10.1016/S0008-6363(02)00735-6 12650867

[B65] Priori S. G. Blomstrom-Lundqvist C. Mazzanti A. Blom N. Borggrefe M. Camm J. (2015). 2015 ESC Guidelines for the management of patients with ventricular arrhythmias and the prevention of sudden cardiac death: the task force for the management of patients with ventricular arrhythmias and the prevention of sudden cardiac death of the European Society of Cardiology (ESC). Endorsed by: Association for European Paediatric and Congenital Cardiology (AEPC). *Eur Heart J.* 36 2793–2867. 10.1093/eurheartj/ehv316 26320108

[B66] Ravens U. Cerbai E. (2008). Role of potassium currents in cardiac arrhythmias. *Europace* 10 1133–1137. 10.1093/europace/eun193 18653669

[B67] Sacconi L. Ferrantini C. Lotti J. Coppini R. Yan P. Loew L. M. (2012). Action potential propagation in transverse-axial tubular system is impaired in heart failure. *Proc. Natl. Acad. Sci. U.S.A.* 109 5815–5819. 10.1073/pnas.1120188109 22451916 PMC3326470

[B68] Sato D. Xie L. H. Sovari A. A. Tran D. X. Morita N. Xie F. (2009). Synchronization of chaotic early afterdepolarizations in the genesis of cardiac arrhythmias. *Proc. Natl. Acad. Sci. U.S.A.* 106 2983–2988. 10.1073/pnas.0809148106 19218447 PMC2651322

[B69] Sequeira V. Wijnker P. J. Nijenkamp L. L. Kuster D. W. Najafi A. Witjas-Paalberends E. R. (2013). Perturbed length-dependent activation in human hypertrophic cardiomyopathy with missense sarcomeric gene mutations. *Circ. Res.* 112 1491–1505. 10.1161/CIRCRESAHA.111.300436 23508784 PMC3675884

[B70] Shan J. Betzenhauser M. J. Kushnir A. Reiken S. Meli A. C. Wronska A. (2010). Role of chronic ryanodine receptor phosphorylation in heart failure and beta-adrenergic receptor blockade in mice. *J. Clin. Invest.* 120 4375–4387. 10.1172/JCI37649 21099115 PMC2993577

[B71] Shannon T. R. Pogwizd S. M. Bers D. M. (2003). Elevated sarcoplasmic reticulum Ca2 + leak in intact ventricular myocytes from rabbits in heart failure. *Circ. Res.* 93 592–594. 10.1161/01.RES.0000093399.11734.B3 12946948

[B72] Sicouri S. Belardinelli L. Antzelevitch C. (2013). Antiarrhythmic effects of the highly selective late sodium channel current blocker GS-458967. *Heart Rhythm* 10 1036–1043. 10.1016/j.hrthm.2013.03.023 23524321 PMC3836836

[B73] Song L. S. Sobie E. A. McCulle S. Lederer W. J. Balke C. W. Cheng H. (2006). Orphaned ryanodine receptors in the failing heart. *Proc. Natl. Acad. Sci. U.S.A.* 103 4305–4310. 10.1073/pnas.0509324103 16537526 PMC1449688

[B74] Sotgia B. Sciagra R. Olivotto I. Casolo G. Rega L. Betti I. (2008). Spatial relationship between coronary microvascular dysfunction and delayed contrast enhancement in patients with hypertrophic cardiomyopathy. *J. Nucl. Med.* 49 1090–1096. 10.2967/jnumed.107.050138 18552138

[B75] Taggart P. Sutton P. Chalabi Z. Boyett M. R. Simon R. Elliott D. (2003). Effect of adrenergic stimulation on action potential duration restitution in humans. *Circulation* 107 285–289. 10.1161/01.CIR.0000044941.13346.74 12538429

[B76] Terracciano C. M. Philipson K. D. MacLeod K. T. (2001). Overexpression of the Na^+^/Ca^2+^ exchanger and inhibition of the sarcoplasmic reticulum Ca^2+^-ATPase in ventricular myocytes from transgenic mice. *Cardiovasc. Res.* 49 38–47. 10.1016/S0008-6363(00)00205-4 11121794

[B77] Terrenoire C. Clancy C. E. Cormier J. W. Sampson K. J. Kass R. S. (2005). Autonomic control of cardiac action potentials: role of potassium channel kinetics in response to sympathetic stimulation. *Circ. Res.* 96 e25–e34. 10.1161/01.RES.0000160555.58046.9a 15731462

[B78] Toischer K. Rokita A. G. Unsold B. Zhu W. Kararigas G. Sossalla S. (2010). Differential cardiac remodeling in preload versus afterload. *Circulation* 122 993–1003. 10.1161/CIRCULATIONAHA.110.943431 20733099 PMC2955196

[B79] Ulus T. Kudaiberdieva G. Gorenek B. (2013). The onset mechanisms of ventricular tachycardia. *Int. J. Cardiol.* 167 619–623. 10.1016/j.ijcard.2012.09.034 23040077

[B80] van der Velden J. Ho C. Y. Tardiff J. C. Olivotto I. Knollmann B. C. Carrier L. (2015). Research priorities in sarcomeric cardiomyopathies. *Cardiovasc. Res.* 105 449–456. 10.1093/cvr/cvv019 25631582 PMC4375392

[B81] Wagner S. Dybkova N. Rasenack E. C. Jacobshagen C. Fabritz L. Kirchhof P. (2006). Ca^2+^/calmodulin-dependent protein kinase II regulates cardiac Na^+^ channels. *J. Clin. Invest.* 116 3127–3138. 10.1172/JCI26620 17124532 PMC1654201

[B82] Wagner S. Hacker E. Grandi E. Weber S. L. Dybkova N. Sossalla S. (2009). Ca/calmodulin kinase II differentially modulates potassium currents. *Circ. Arrhythm Electrophysiol.* 2 285–294. 10.1161/CIRCEP.108.842799 19808479 PMC2734104

[B83] Weisser-Thomas J. Piacentino V. III Gaughan J. P. Margulies K. Houser S. R. (2003). Calcium entry via Na/Ca exchange during the action potential directly contributes to contraction of failing human ventricular myocytes. *Cardiovasc. Res.* 57 974–985. 10.1016/S0008-6363(02)00732-0 12650875

[B84] Xu L. Lai D. Cheng J. Lim H. J. Keskanokwong T. Backs J. (2010). Alterations of L-type calcium current and cardiac function in CaMKII{delta} knockout mice. *Circ. Res.* 107 398–407. 10.1161/CIRCRESAHA.110.222562 20538682 PMC2923749

[B85] Yang B. Lin H. Xiao J. Lu Y. Luo X. Li B. (2007). The muscle-specific microRNA miR-1 regulates cardiac arrhythmogenic potential by targeting GJA1 and KCNJ2. *Nat. Med.* 13 486–491. 10.1038/nm1569 17401374

